# A Positive Feedback Loop of lncRNA DSCR8/miR-98-5p/STAT3/HIF-1α Plays a Role in the Progression of Ovarian Cancer

**DOI:** 10.3389/fonc.2020.01713

**Published:** 2020-09-02

**Authors:** Lina Dong, Xuejiao Cao, Yi Luo, Guoqing Zhang, Dandan Zhang

**Affiliations:** ^1^Department of Obstetrics and Gynecology Ultrasound, The First Affiliated Hospital of Harbin Medical University, Harbin, China; ^2^Department of Obstetrics and Gynecology, The First Affiliated Hospital of Harbin Medical University, Harbin, China

**Keywords:** ovarian cancer, DSCR8, miR-98-5p, signal transducer and activator of transcription 3, hypoxia inducible factor 1 alpha

## Abstract

**Background:**

Accumulating studies have revealed that long non-coding RNA (lncRNA) and microRNA (miRNA) contribute to ovarian cancer (OC). DSCR8 has been found to mediate hepatocellular carcinoma development, while its role in OC remains to be explored.

**Methods:**

In this study, lncRNA DSCR8 and miR-98-5p expressions in OC tissues and adjacent non-cancer tissues were determined by reverse transcriptase polymerase chain reaction (RT-PCR). Besides, gain-of-function or loss-of-function assays of DSCR8 and miR-98-5p were conducted on OC cell lines SKOV-3 and A2780. Cell proliferation was detected with Cell Counting Kit (CCK)8 and colony formation assay, and western blot was used to test the apoptotic levels of OC cells. Transwell assay was conducted to examine cell invasion, and the epithelial–mesenchymal transition (EMT) of OC cells was tested by western blot. Moreover, luciferase activity assay and RNA immunoprecipitation (RIP) assay were conducted to verify the relationships between DSCR8 and miR-98-5p, miR-98-5p, and signal transducer and activator of transcription 3 (STAT3).

**Results:**

DSCR8 was remarkedly increased in OC tissues and associated with poorer survival of OC patients. Overexpressing DSCR8 promoted cell proliferation, invasion, and EMT but inhibited apoptosis. On the other hand, miR-98-5p was downregulated in OC tissues and relieved the progression of OC. Moreover, overexpressed DSCR8 increased the levels of STAT3 and hypoxia inducible factor 1 alpha (HIF-1α) and dampened the functions of miR-98-5p on OC. Pharmaceutical intervention of STAT3 and HIF-1α significantly altered the expressions of DSCR8 and miR-98-5p.

**Conclusion:**

The present results suggested a positive feedback loop of lncRNA DSCR8/miR-98-5p/STAT3/HIF-α axis in the progression of OC.

## Background

Ovarian cancer (OC) is a common malignancy of the female genital system, ranking next to cervical cancer and endometrial cancer (1). Due to its occult onset and intense metastasis, the early diagnosis of OC is challenging (2). Therefore, exploring the molecular mechanism of OC is of great significance for its early diagnosis and therapy.

Long non-coding RNAs (lncRNAs) are a class of non-coding RNAs widely existing in cells, with lengths over 200 bp. Although lncRNAs cannot encode a protein, they exert remarkable roles in regulating tumor progression (3, 4). Functionally, lncRNAs are widely involved in tumor proliferation, apoptosis, cell cycle, immunity, and drug tolerance (5, 6). Therefore, abnormally expressed lncRNAs can be used as a prognostic diagnostic marker and a therapeutic target for tumors (6). It is noteworthy that increasing studies have indicated that lncRNAs play a significant role in the development of OC. For example, lncRNA LINC00565 (7) and lncRNA SNHG14 (8) are upregulated in OC and promote its development. In contrast, lncRNA GAS5 (9) and lncRNA LINC01125 (10) have antitumor effects in OC. On the other hand, lncRNA DSCR8 has been found overexpressed in hepatocellular carcinoma (HCC) and promotes proliferation and migration of HCC cells by regulating miR-485-5p and Wnt/β-catenin signaling pathway (11). Nevertheless, the role of lncRNA DSCR8 in OC is limited.

Meanwhile, microRNAs (miRNAs) are another kind of non-coding RNAs, which are about 18–22 bp in length. By binding to the mRNAs 3′-untranslated region (UTR) end, miRNAs modulate gene expression at the posttranscriptional level (12). Interestingly, multiple miRNAs have also been found abnormally expressed in tumors and regulate their cell biological processes (13). MiR-98-5p is one member of miRNAs, and its downregulation has been shown to promote pancreatic ductal adenocarcinoma (PDAC) proliferation and metastasis (14). In addition, it is found that miR-98-5p enhances the sensitivity of epithelial OC to cisplatin (15). However, the mechanism of miR-98-5p in regulating OC needs further exploration.

Signal transducer and activator of transcription 3 (STAT3) is one of the crucial members of Janus kinase (JAK)/STAT pathways. Studies have found that miRNA cluster MC-let-7a-1∼let-7d regulates glioma autophagy and apoptosis by targeting STAT3 (16). Besides, hypoxia inducible factor 1 alpha (HIF-1α), a prime modulator of cellular and systemic homeostatic response to hypoxia, can be activated by STAT3 and then regulate tumorigenesis (17). Here, our previous study demonstrates that lncRNA DSCR8 is upregulated in OC. Then we conducted gain-of-function and loss-of-function experiments and found that lncRNA DSCR8 promoted the proliferation and metastasis while inhibiting apoptosis of OC cells. Moreover, lncRNA DSCR8 has been found to upregulate STAT3 and HIF-1α. Further, the bioinformatics analysis revealed that miR-98-5p shares the binding sites with lncRNA DSCR8 and STAT3. Therefore, we speculate that there is an lncRNA DSCR8/miR-98-5p/STAT3/HIF-α axis on regulating the progression of OC.

## Materials and Methods

### Clinical Sample Selection

Tumor and adjacent normal tissues (>3 cm away from cancer tissues) of the patients were collected, and the patients were diagnosed with OC and received surgical treatment in The First Affiliated Hospital of Harbin Medical University from June 2013 to June 2015. All of the tumor and adjacent normal tissues were diagnosed by two experienced pathologists. The Medical Ethics Committee of The First Affiliated Hospital of Harbin Medical University approved this study, and written consents were obtained from all patients involved in the study.

### Cell Culture

The human normal ovarian epithelial cell line IOSE80 and the human OC cell lines A2780, COV644, OV-90, OVCAR-3, As2O3, and SKOV3 were all purchased from the American Type Culture Collection (ATCC, Rockville, MD, United States). DMEM-F12 medium (Thermo Fisher Scientific, Shanghai, China) containing 10% fetal bovine serum (FBS) and 1% penicillin and streptomycin were used for cell culture. When the cells were 80% confluent, 0.25% trypsin (Thermo Fisher HyClone, Utah, UT, United States) was used for cell trypsinization and passage. Cells at the logarithmic growth phase were selected for later use.

### Cell Transfection and Treatment

DSCR8 overexpressing plasmids, DSCR8 short-hairpin RNA (sh-DSCR8), as well as miR-98-5p mimics and their negative controls were all designed and synthesized by Shanghai Integrated Biotech Solutions Co., Ltd. Twenty-four-well plates were used for culturing SKOV-3 and A2780 cells (1 × 10^5^/well). When the cells were at the logarithmic growth phase, SKOV-3 and A2780 cells were transfected with the above expressing vectors with Lipofectamine^®^ 3000 (Invitrogen; Thermo Fisher Scientific, Inc.) according to the manufacturer’s instructions. Twenty-four hours after transfection, the original culture medium was discarded and replaced with fresh DMEM-F12 complete medium for 48 h. Reverse transcriptase polymerase chain reaction (RT-PCR) was used to verify the transfection efficiency of DSCR8 and miR-98-5p in cells.

signal transducer and activator of transcription 3-specific inhibitor Stattic (Cat. No. HY-13818) and HIF-1α specific inhibitor BAY 87-2243 (Cat. No. HY-15836) were purchased from MedChemExpress (Shanghai, China). When the cells were in the logarithmic growth phase, 20 μM of Stattic and 20 nM of BAY 87-2243 were applied to treat OC cells for 24 h to interfere with the activation of STAT3 or HIF-1α.

### Cell Counting Kit Assay

SKOV-3 and A2780 cells in the logarithmic growth phase were trypsinized and adjusted to 2 × 10^3^/ml, then inoculated in 96-well plates with 100 μl cell suspension per well. Each group set three repetitive wells. Afterward, the 96-well plates were placed in an incubator for further culture. After 24 h, each well was supplied with 10 μl of Cell Counting Kit (CCK)8 liquor (Hubei Bios Biotechnology Co., Ltd.), and the cells were incubated for one more hour. After that, a microplate reader was adopted to measure the absorbance (OD value) at 450 nm wavelength of 96-well plates. Thereafter, the OD values of the cells were measured once at 24, 48, 72, and 96 h.

### Colony Formation Assay

SKOV-3 and A2780 cells in the logarithmic phase were selected and inoculated in 60-mm dishes containing culture medium with 100 cells per dish, then the plates were placed in the incubator. The cells were cultured for 12 days in a medium that was altered every 3–4 days. Then, phosphate buffered saline (PBS) was used to wash cells, and 4% paraformaldehyde was adopted for cell fixing. Afterward, cells were stained with crystal violet and photographed. Finally, Image-ProPlus was used to calculate the number of cell colonies in each dish.

### RT-PCR

The relative expressions of DSCR8 and miR-98-5p in tissues and cells were tested by RT-PCR. TRIzol reagent (Invitrogen, Carlsbad, CA, United States) was used to extract total RNA, and RevertAid First Strand cDNA Synthesis Kit (Thermo Fisher Scientific, Waltham, MA, United States) was adopted to reversely transcribe RNA into cDNA after its purity was measured by a UV spectrophotometer. PCR amplification was conducted with TOYOBO SYBR Green Realtime PCR Master Mix (TOYOBO, Osaka, Japan) by using cDNA as a template. The PCR reaction conditions were as follows: 40 cycles at 95°C for 30 s, 95°C for 5 s, 60°C for 30 s, and 73°C for 10 s. The endogenous control of DSCR8 and STAT3 was glyceraldehyde 3-phosphate dehydrogenase (GAPDH), while that of miR-98-5p was U6. The 2^–ΔΔCT^ method was adopted to count the relative expression of DSCR8 and miR-98-5p. The sequences of each primer were as follows: DSCR8, Forward primer, 5′-CTCCACCTCCCAGTTCAAGA-3′, Reverse primer, 5′-CACGGCATGAACTGAATGGA-3′; STAT3, Forward primer, 5′-AGAAGGAGGCGTCACTTTCA-3′, Reverse primer, 5′-TTTCCGAATGCCTCCTCCTT-3′; miR-98-5p, Forward primer, 5′-TGAGGTAGTAGTTTGTGCTGTT-3′, miRNA universal primer, 5′-GCGAGCACAGAATTAATACGAC-3′; GAPDH, Forward primer, 5′-CTGGGCTACACTGAGCACC-3′, Reverse primer, 5′-AAGTGGTCGTTGAGGGCAATG-3′; U6, Forward primer, 5′-CTCGCTTCGGCAGCACA-3′, Reverse primer, 5′-AACGCTTCACGAATTTGCGT-3′.

### Western Blot

The total protein of the cells in the experimental group and the control group was extracted. The concentration of the proteins was determined by the bicinchoninic acid (BCA) method. Next, sodium dodecyl sulfate (SDS)-polyacrylamide gel electrophoresis (SDS-PAGE) was used to separate the proteins, which were then electrically transferred into the polyvinylidene fluoride (PVDF) membranes at a constant current of 300 mA. Afterward, the PVDF membranes were sealed with a Tris-buffered saline–Tween (TBST) solution which contained 5% non-fat milk at room temperature for 1 h. Blocking buffer was used to dilute the following primary antibodies of protein incubated with the sealed PVDF membrane at 4°C overnight: STAT3 (Abcam, ab119352, 1:1,200), STAT3 (phospho Y705) (Abcam, ab76315, 1:1,000), JAK2 (Abcam, ab18596, 1:1,000), JAK2 (phospho Y1007 + Y1008) (Abcam, ab32101, 1:1,200), HIF-1α (Abcam, ab51608, 1:1,000), Bax (Abcam, ab32503, 1:1,000), Bcl2 (Abcam, ab32124, 1:1,000), Caspase3 (Abcam, ab13847, 1:1,000), E-cadherin (Abcam, ab1416, 1:1,000), Vimentin (Abcam, ab8978, 1:1,000), and N-cadherin (Abcam, ab18203, 1:1,000), then the membranes were washed with PBST for four times with 8 min each. Subsequently, the membranes were incubated with the corresponding secondary antibodies (dilution concentration 1:2,000) for 1.5 h at room temperature before the membranes were rinsed with TBST. X-ray development was performed using Pierce ECL western blot Substrate kit from Thermo Corporation.

### *In vivo* Experiment

Twenty 4–6 weeks old BALB/c nude mice were randomly split into four groups. OC cells were transfected with DSCR8 overexpressing plasmids and the negative control (NC) and sh-DSCR8 and its negative control (sh-NC). Stably transfected OC cells were chosen and adjusted to 2 × 10^8^ ml^–1^. Then, 0.1 ml of the cell suspension was injected subcutaneously into the axilla of the left forelimb of nude mice. The mouse survival rate, weight, and status were counted within 4 weeks after the injection, and tumor size and weight were measured.

### Dual-Luciferase Reporter Gene Assay

All luciferase reporter vectors (DSCR8-wt, DSCR8-mt, STAT3-wt, and STAT3-mt) were constructed by Promega Corporation (Promega, Madison, WI, United States). The segments of DSCR8-mt and STAT3-mt vectors containing miR-98-5p binding sites were mutated. OC cells SKOV-3 and A2780 (4.5 × 10^4^) were seeded in 48-well plates, cultured to 70% confluence, and then co-transfected with DSCR8-wt, DSCR8-mt, STAT3-wt, and STAT3-mt and miR-98-5p mimics or negative control using Lipofectamine^®^ 3000 (Invitrogen; Thermo Fisher Scientific, Inc.), respectively. Luciferase activity was tested according to the manufacturer’s guidance. All experiments were in triplicate.

### RNA Immunoprecipitation Assay

Ovarian cancer cells SKOV-3 and A2780 were transfected with miR-98-5p mimic and its negative control (NC-mimic), respectively, then collected and added into RNA immunoprecipitation (RIP) lysis buffer for cell lysis. Afterward, RIP buffer containing magnetic beads conjugated to a human anti-Ago2 antibody (Millipore, United States) was used to culture cell extraction. Samples were with proteinase K with shaking to trypsinize proteins, and the immunoprecipitated RNA was isolated. Subsequently, RNA concentration was determined by the Drop spectrophotometer. DSCR8 and STAT3 levels in the purified RNA were subjected to RT-PCR analysis.

### Data Statistics

SPSS 22.0 (SPSS Inc., Chicago, IL, United States) was used for data processing. The normally distributed data were expressed as mean ± standard deviation ($\bar{x}$ ± *s*), and *t*-test was used for pairwise comparison. Comparison between multiple groups was analyzed by one-way analysis of variance, and multiple comparisons between groups were performed by LSD-t method. *P* < 0.05 was statistically significant.

## Results

### The Expression Characteristics of DSCR8 and miR-98- 5p in Ovarian Cancer Tissues

**FIGURE 1:**
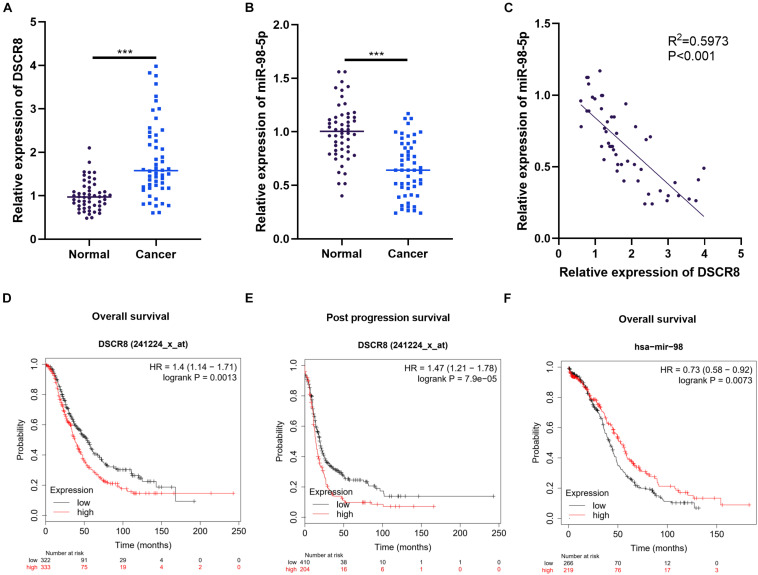
Expression characteristics of DSCR8 and miR-98-5p in ovarian cancer (OC) tissues. **(A–C)** RT-PCR was used to examine the expressions of DSCR8 and miR-98-5p in 52 OC and adjacent normal tissues. It was revealed that DSCR8 was distinctly upregulated **(A)**, while miR-98-5p was significantly downregulated **(B)** compared with that of normal adjacent tissues, ***P < 0.001. Linear regression analysis showed that DSCR8 negatively correlates to miR-98-5p expression **(C)**. **(D–F)** The diagnostic significance of DSCR8 and miR-98-5p expression levels on prognosis was analyzed through KM plotter (http://kmplot.com/analysis/index.php?p=service). DSCR8 was significantly associated with overall survival **(D)** and post progression survival **(E)** in patients with cervical cancer. MiR-98-5p is strongly associated with overall survival **(F)** in OC patients.

First, we examined DSCR8 and miR-98-5p expressions in OC tissues by RT-PCR. It was found that DSCR8 was obviously upregulated (Figure 1A), while miR-98-5p was distinctly downregulated (Figure 1B) in OC tissues compared with that in adjacent normal tissues. Further analysis confirmed that DSCR8 and miR-98-5p expressions were negatively correlated (Figure 1C). In addition, we analyzed the diagnostic roles of DSCR8 and miR-98-5p in the prognosis of OC patients by KM plotter^1^, a system containing gene chip and RNA-seq data sources for the databases include Gene Expression Omnibus (GEO), European Genome-phenome Archive (EGA), and The Cancer Genome Atlas (TCGA). The results indicated that both DSCR8 upregulation and miR-98-5p downregulation were closely related to OC patients’ poor prognosis (Figures 1D–F). Moreover, we analyzed the correlation between the clinical pathological indicators and DSCR8 and miR-98-5p levels in 52 patients with OC. The results turned out that patients with a high level of DSCR8 had significantly increased tumor volume and International Federation of Gynecology and Obstetrics (FIGO) stage and were more prone to distant metastasis, and patients with a low expression of miR-98-5p also had noticeable elevated tumor volume and FIGO stage (Tables 1, 2). Overall, the above data strongly suggested that DSCR8 and miR-98-5p make a significant contribution to OC progression.

**TABLE 1:** Correlation between clinicopathological features and DSCR8 expression in OC tissues.

Clinicopathologic features	Level of DSCR8		*P*-value
	High (*n* = 26)	Low (*n* = 26)	
**Age**			
<50 years	10	8	0.5599
≥50 years	16	18	
**Histology**			
Serous	17	14	0.3965
Others	9	12	
**Differentiation**			
Well to moderate	16	19	0.3751
Poor	10	7	
**FIGO stage**			
I–II	11	19	0.0247
III–IV	15	7	
**Lymph node metastasis**			
Yes	12	4	0.0162
No	14	22	
**Tumor size**			
>5 cm	15	8	0.05
≤ 5 cm	11	18	

**TABLE 2:** Correlation between clinicopathological features and miR-98-5p expression in OC tissues.

Clinicopathologic features	Level of miR-98-5p		*P*-value
	High (*n* = 26)	Low (*n* = 26)	
**Age**			
<50 years	11	7	0.2436
≥ 50 years	15	19	
**Histology**			
Serous	16	15	0.7774
Others	10	11	
**Differentiation**			
Well to moderate	20	15	0.1394
Poor	6	11	
**FIGO stage**			
I–II	22	8	<0.001
III–IV	4	18	
**Lymph node metastasis**			
Yes	7	9	0.5479
No	19	17	
**Tumor size**			
>5 cm	7	16	0.012
≤ 5 cm	19	10	

### DSCR8 Enhanced the Development of Ovarian Cancer *in vitro*

**FIGURE 2:**
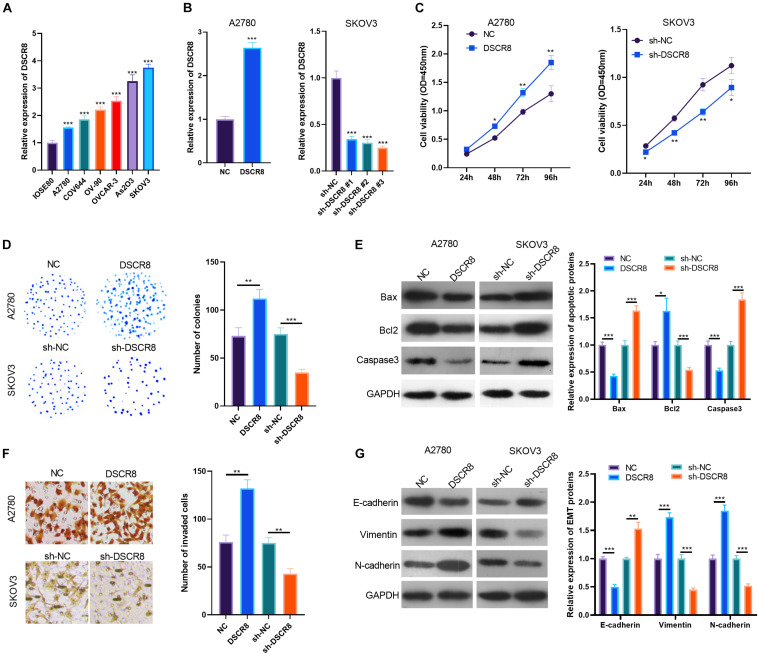
DSCR8 promoted ovarian cancer (OC) progression *in vitro*. **(A)** RT-PCR was adopted to test DSCR8 expressions in human normal ovarian epithelial cell line IOSE80 and human OC cell lines A2780, COV644, OV-90, OVCAR-3, As2O3, and SKOV3. ***P < 0.001 compared with the IOSE80 group. **(B)** DSCR8 overexpressing and knocking down cell models were respectively constructed in A2780 and SKOV-3 cells. **(C,D)** Cell proliferation was tested by Cell Counting Kit (CCK)8 assay **(C)** and colony formation assay **(D)**. *P < 0.05, **P < 0.01 vs. NC or sh-NC group, respectively. **(E)** Apoptosis-related proteins (Bax, Bcl2, and Caspase3) expressions were tested by western blot. **(F)** Transwell was used for detecting OC cell invasion. **(G)** Western blot was used to detect epithelial–mesenchymal transition (EMT) markers (E-cadherin, N-cadherin, and Vimentin) expression. *P < 0.05, **P < 0.01, ***P < 0.001.

To explore the effects of DSCR8 on OC progression, firstly, we examined DSCR8 expression in the human normal ovarian epithelial cell line IOSE80 and the human OC cell lines A2780, COV644, OV-90, OVCAR-3, As2O3, and SKOV3. The results manifested that the DSCR8 was overexpressed in OC cells compared with IOSE80 cells (Figure 2A). Next, we constructed DSCR8 overexpressing and knocking down cell model in A2780 and SKOV-3, respectively (Figure 2B). Meanwhile, we carried out CCK8 and colony formation experiments to test cell proliferation. The results showed that overexpressing DSCR8 dramatically promoted the proliferation of A2780, while knocking down DSCR8 inhibited the cell growth (Figures 2C,D). Further, we used western blot to detect apoptotic related proteins. The results revealed that overexpressing DSCR8 decreased Bax and Caspase expression and enhanced Bcl2 expression, while knocking down DSCR8 had the opposite effects (Figure 2E). In addition, we examined the invasion and epithelial–mesenchymal transition (EMT) of OC cells. It was found that overexpressing DSCR8 notably promoted the invasive capacity and EMT of OC cells. Nevertheless, knocking down DSCR8 hampered the invasion and EMT of OC cells (Figures 2F,G). Overall, these data indicated that DSCR8 enhances OC proliferation, invasion, and EMT and inhibits apoptosis.

### DSCR8 Promoted Tumor Growth and Migration *in vivo*

**FIGURE 3:**
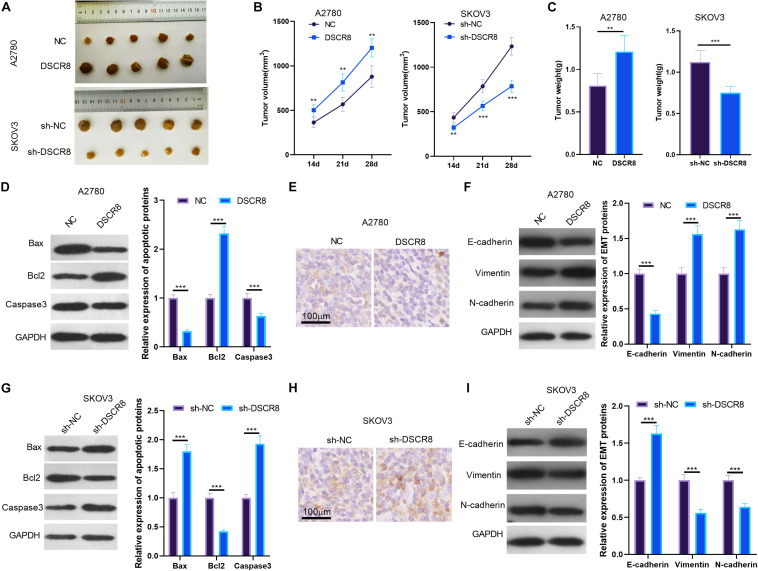
DSCR8 promoted tumor growth and epithelial–mesenchymal transition (EMT) *in vivo*. A2780 with overexpressed DSCR8 and SKOV-3 with knocked down DSCR8 were injected into nude mice. Four weeks later, tumor tissues from nude mice were isolated. **(A)** Tumor images of each group. **(B)** Tumor volume of each group. **P < 0.01, ***P < 0.001 vs. NC or sh-NC group, respectively. **(C)** Tumor weight of each group. **(D,G)**. Western blot was used to measure the apoptosis (Bax, Bcl2, and Caspase3) in the formed tissues. **(E,F,H,I)**. Immunohistochemistry and western blot were used to detect EMT-related proteins (E-cadherin, Vimentin, and N-cadherin) levels in tumor tissues. ***P < 0.001.

To further confirm the functions of DSCR8 on cancer growth, we established the A2780 cell model with overexpressing DSCR8 and SKOV-3 with knocking down DSCR8 and conducted tumor xenograft in nude mice. After 4 weeks, the tumor tissues were isolated. It was found that overexpressing DSCR8 notably promoted tumor growth, while knocking down DSCR8 dampened tumor growth (Figures 3A–C). Furthermore, we detected apoptosis and EMT-related protein expression in tumor tissues. Interestingly, we found that Bax and Caspase3 expression levels were markedly decreased, while BCl2 was increased in the DSCR8 overexpressed group. In contrast, Bax and Caspase3 levels were elevated, while that of BCl2 was remarkably reduced in the sh-DSCR8 group (Figures 3D,G). In addition, overexpressing DSCR8 attenuated the expression of E-cadherin, an epithelial cell marker, and promoted mesenchymal cell markers of Vimentin and N-cadherin, while knocking down DSCR8 inhibited EMT (Figures 3E,F,H,I). These results further demonstrated that DSCR8 promotes tumor growth and metastasis.

### DSCR8 Targeted MiR-98-5p

**FIGURE 4:**
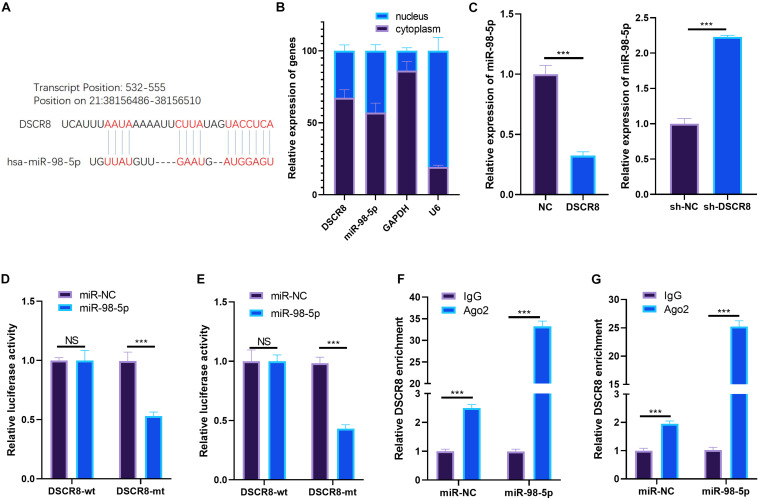
DSCR8 targeted miR-98-5p. **(A)** Through lncBase v.2 (http://carolina.imis.athena-innovation.gr/diana_tools/web/index.php?r=lncbasev2%2Findex), we predicted the targets of long non-coding RNA (lncRNA) DSCR8 and found that miR-98-5p is one of the targets of DSCR8. **(B)** RT-PCR was used to detect DSCR8 and miR-98-5p in the cytoplasm and nucleus. **(C)** RT-PCR was adopted for monitoring miR-98-5p, which selectively regulates DSCR8 in OC cells. **(D,E)** Dual luciferase reporter assay was performed to verify miR-98-5p and DSCR8 correlation. **(F,G)** The relationship between miR-98-5p and DSCR8 was verified by RNA immunoprecipitation (RIP) experiments. NS = *P* > 0.05, ***P < 0.001.

Studies have revealed that lncRNAs sponge corresponding miRNA as a competitive endogenous RNA (ceRNA). Here, we were also curious about the ceRNAs of DSCR8. Through LncBase Predicted v.2^2^, we found that miR-98-5p is one ceRNA of DSCR8 (Figure 4A). By detecting the distribution of DSCR8 and miR-98-5p in OC cells, we found that both DSCR8 and miR-98-5p were mainly expressed in the cytoplasm of OC cells (Figure 4B). Moreover, the miR-98-5p expression in DSCR8 selectively regulated OC cells was examined by RT-PCR. It was found that overexpressing DSCR8 obviously inhibited miR-98-5p expression, while knocking down DSCR8 upregulated miR-98-5p (Figure 4C). In order to further clarify the binding relationship between DSCR8 and miR-98-5p, dual luciferase reporter assay was conducted. The results showed that miR-98-5p mimics inhibited the luciferase of cells transfected with DSCR8-wt but had no significant effects on that with DSCR8-mt. Besides, the RIP experiment revealed that DSCR8 was more significantly enriched in both A2780 and SKOV-3 cells transfected with miR-98-5p (Figures 4D–G). Consequently, those data suggested that DSCR8 targeted and negatively regulated miR-98-5p expression in OC.

### DSCR8 Attenuated the Antitumor Effects of MiR-98-5p on Ovarian Cancer Cells

**FIGURE 5:**
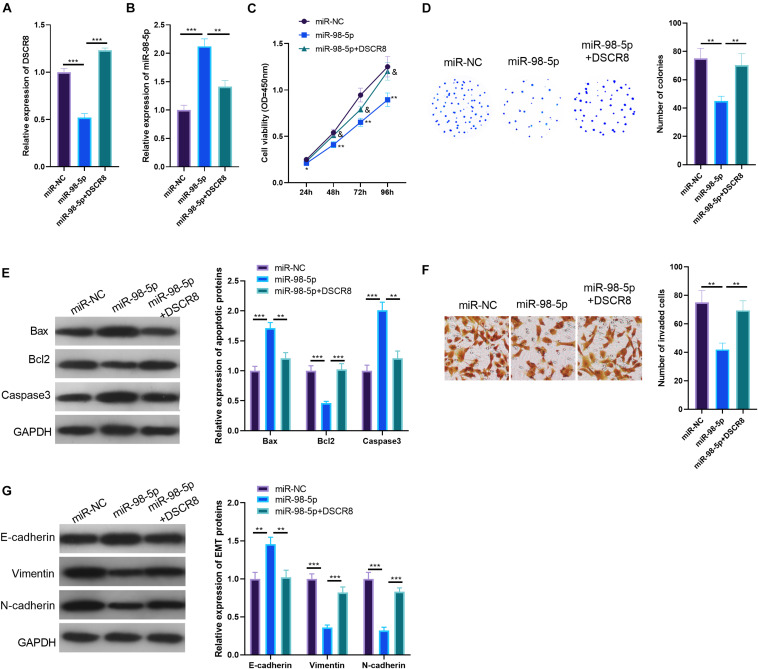
DSCR8 weakened the effects of miR-98-5p. **(A,B)** RT-PCR was conducted to determine the expression of DSCR8 **(A)** and miR-98-5p **(B)**. **(C)** Cell Counting Kit (CCK)8 was performed for cell proliferation detection. *P < 0.05, **P < 0.01 vs. miR-NC group; & represents *P* < 0.05 vs. miR-98-5p group. **(D)** Cell growth was further verified by colony formation test. **(E)** Western blot was used to monitor apoptosis-related proteins (Bax, Bcl2, and Caspase3) expression. **(F)** Transwell assay was used to detect ovarian cancer (OC) cell invasion. **(G)** Western blot was used to observe epithelial–mesenchymal transition (EMT) markers (E-cadherin and Vimentin) expressions.**P < 0.01, ***P < 0.001.

To investigate the regulatory role of DSCR8/miR-98-5p in OC cells, we constructed a miR-98-5p-overexpressing cell model in A2780 cell. On this basis, we further transfected A2780 cells with DSCR8 overexpressing plasmids. Our results of RT-PCR indicated that DSCR8 was inhibited by miR-98-5p mimics (compared with the miR-NC group). Meanwhile, miR-98-5p was noticeably downregulated after overexpression of DSCR8 (compared with the miR-98-5p group) (Figures 5A,B). Furthermore, CCK8 assay, colony formation experiment, Transwell assay, and western blot were adopted to verify cell proliferation, apoptosis, invasion, and EMT. The results validated that overexpressing miR-98-5p dramatically inhibited the OC cell proliferation (Figures 5C,D), invasion (Figure 5F), and EMT (Figure 5G) while promoting apoptosis (Figure 5E). On the other hand, overexpressing DSCR8 markedly weakened the above effects (compared with the miR-98-5p group) (Figures 5C–G). Therefore, miR-98-5p is a tumor-suppressive gene in OC, and DSCR8 promotes the progression of OC by inhibiting miR-98-5p.

### The Effects of DSCR8/MiR-98-5p on Signal Transducer and Activator of Transcription 3/Hypoxia Inducible Factor 1 Alpha Pathway

**FIGURE 6:**
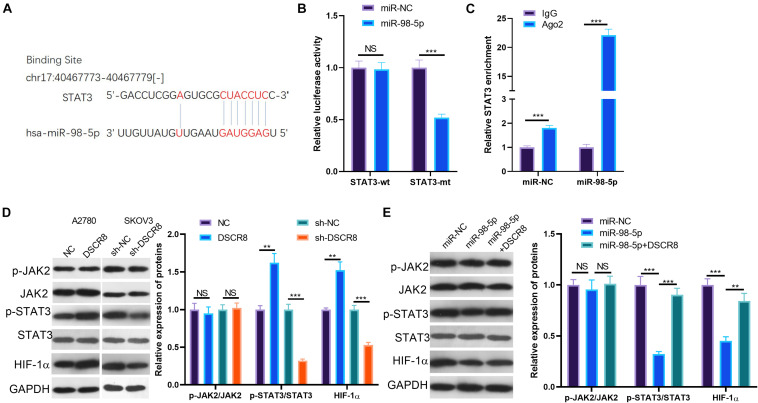
DSCR8/miR-98-5p regulation on signal transducer and activator of transcription 3 (STAT3)/hypoxia inducible factor 1 alpha (HIF-1α). **(A)** Through TargetScan database (http://www.targetscan.org/vert_72/), it was found that miR-98-5p targets STAT3. **(B,C)** The results of the dual luciferase reporter assay **(B)** and the RNA immunoprecipitation (RIP) assay **(C)** confirmed that miR-98-5p targeted the 3′-untranslated region (3′UTR) end of the STAT3 mRNA **(D)**. **(D,E)** Janus kinase (JAK)2/STAT3/HIF-1α pathway expression was determined by western blot. NS = *P* > 0.05, **P < 0.01, ***P < 0.001.

Janus kinase 2/signal transducer and activator of transcription 3/hypoxia inducible factor 1 alpha is a classic signaling pathway, which plays a significant role in regulating tumors. In this study, our results of bioinformatics analysis found that miR-98-5p is a novel target on STAT3 (Figure 6A). To identify the relationship between miR-98-5p and STAT3, we performed dual luciferase reporter assay and RIP experiment, and the results verified that miR-98-5p targeted the 3′-UTR of STAT3 (Figures 6B,C). Further, we tested the expression of the JAK2/STAT3/HIF-1α pathway after regulating DSCR8 and miR-98-5p. The results showed that overexpressing DSCR8 increased the levels of phosphorylated STAT3 and HIF-1α, while downregulating DSCR8 or enhancing miR-98-5p expression inhibited the expression of STAT3 and HIF-1α (Figures 6D,E). These results manifested that DSCR8 promotes STAT3/HIF-1α activation through miR-98-5p.

### Suppression of Signal Transducer and Activator of Transcription 3/Hypoxia-Inducible Factor-1 Alpha Inhibited DSCR8 Expression

**FIGURE 7:**
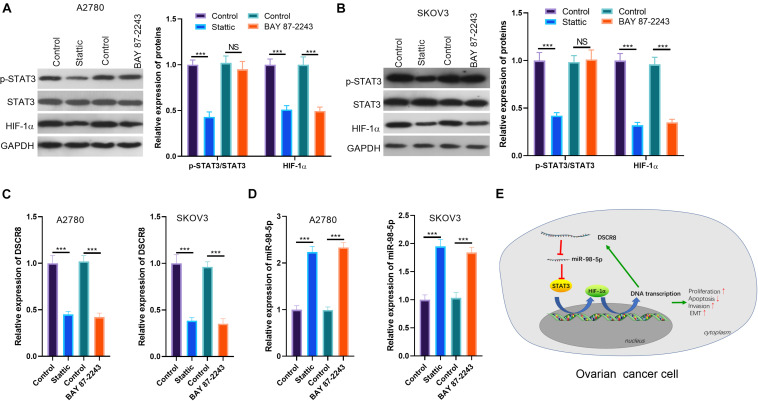
Signal transducer and activator of transcription 3 (STAT3)/hypoxia inducible factor 1 alpha (HIF-1α) inhibition dampened DSCR8 expression. **(A,B)** Ovarian cancer (OC) cells were respectively treated with 20 μM of Stattic and 20 nM of BAY 87-2243 to intervene STAT3 or HIF-1α for 24 h. Western blot was used for Janus kinase (JAK)2/STAT3/HIF-1α detection. **(C,D)** RT-PCR was used to examine DSCR8 and miR-98-5p expressions in cells, respectively. **(E)** The mechanism diagram of positive feedback network DSCR8/miR-98-5p/STAT3/HIF-1α in OC cells. ***P < 0.001.

Our previous studies have confirmed the regulatory effect of DSCR8/miR-98-5p on the STAT3/HIF-1α pathway. Still, we wondered whether STAT3/HIF-1α regulates the expression of DSCR8/miR-98-5p. Therefore, we treated OC cells SKOV-3 and A2780 with the inhibitors of STAT3 and HIF-1α, respectively. Interestingly, we found that Stattic (a STAT3 inhibitor) distinctly reduced STAT3 phosphorylation and HIF-1α expression. On the other hand, BAY 87-2243 (an HIF-1α inhibitor) inhibited the HIF-1α expression, while having no significant effect on the levels of JAK2 and STAT3 (Figures 7A,B). Next, we conducted RT-PCR to detect DSCR8 and miR-98-5p expressions in the cells. It was indicated that DSCR8 was remarkably downregulated in cells after inhibition of STAT3 and HIF-1α (Figure 7C), while miR-98-5p was noticeably upregulated (Figure 7D). Thus, there is a positive feedback axis in OC, namely, the DSCR8/miR-98-5p/STAT3/HIF-1α axis. The overexpressed DSCR8 upregulates the STAT3/HIF-1α pathway by sponging miR-98-5p, which in turn promotes DSCR8 expression (Figure 7E).

## Discussion

The research explored the role of DSCR8 and miR-98-5p in OC progression. We found that DSCR8 was upregulated in OC tissues and predicted poor prognosis of OC patients. Further, *in vivo* and *in vitro* experiments affirmed that DSCR8 promotes OC progression through upregulating the STAT3/HIF-1α pathway by targeting miR-98-5p.

Various genes are abnormally expressed during the progression of OC, and many genes have been found to play important roles in regulating the proliferation, apoptosis, cycle, metastasis, and chemotherapy resistance of OC. As one of the research hot spots in recent years, growing lncRNAs have been discovered, and their role in OC progression has been announced by increasing researches with the development of next-generation sequencing technology. For example, UNC5B-AS1 is upregulated in OC tissues, and overexpressing UNC5B-AS1 enhances proliferation and inhibits apoptosis of OC tissues (18). In addition, lncRNA TUBA4B is downregulated in epithelial OC tissues and is compactly related to the FIGO stage and distant metastasis of OC patients (19). On the other hand, SNHG5 is downregulated in OC tissue, while upregulating SNHG5 improves the sensitivity of OC cells to paclitaxel (20). As a matter of fact, lncRNAs also exert a role in the microenvironment of OC. The differentially expressed lncRNAs not only can be used as distinguishing cancer-associated fibroblasts (CAFs) from normal ovarian fibroblast (NOF) but also modulate the metastasis-promoting phenotype of CAFs (21) and the immune response of CAFs (22). Here we explore the effects of DSCR8 in OC. Similar to previous studies (11), this study discovered that DSCR8 promotes the progression, including cell proliferation and growth, while inhibiting the apoptosis of OC. More importantly, DSCR8 also closely correlates with the poor prognosis of OC patients, indicating that DSCR8 can be used as a target for OC diagnosis and therapy.

As another type of non-coding RNAs, miRNAs regulate tumorigenesis and tumor development by modulating tumor-related gene expression in cells, and this effect also makes a particular contribution to OC development. For example, miR-139 is downregulated in OC tissues and regulates its apoptosis by targeting ATP7A (23). On the other hand, miR-552 targets PTEN, a key tumor suppressive gene, and thus promotes OC growth and metastasis (24). Here, we explored the functions of miR-98-5p on OC. The results revealed that overexpressing miR-98-5p dramatically dampened the proliferation, invasion, and EMT while promoting the apoptosis of OC cells. Interestingly, previous studies have manifested that miR-98-5p is an antitumor gene in PDAC (14), OC (15), and papillary thyroid carcinoma (25), which further confirms our assumption.

Furthermore, miR-98-5p can also be regulated by lncRNA. For example, lncRNA HOXA11-AS upregulates YBX2 and accelerates the proliferation and metastasis of oral squamous cell carcinoma (OSCC) through competitively inhibiting miR-98-5p (26). Further, through bioinformatics analysis, we observed that miR-98-5p is a target of DSCR8, and the targeting relationship between DSCR8 and miR-98-5p was confirmed by dual luciferase reporter assay and RIP experiment. Functionally, forced overexpression of DSCR8 dramatically dampened the antitumor effects induced by miR-98-5p. Collectively, upregulated DSCR8 exerts a carcinogenic effect by inhibiting miR-98-5p in OC.

On the other hand, invasion of OC is an important factor for its distant metastasis. Recent studies have revealed that OC metastasis not only is caused by the passive mechanism of OC cells falling off from the primary tumor but also may be through a variety of signaling pathways (27). As a key step in the process of tumor metastasis, EMT also dramatically affects the metastasis of OC (28). Here, we noticed that DSCR8 and miR-98-5p exert significant effects on the invasion of OC cells. At the same time, overexpressing DSCR8 elevates EMT, while miR-98-5p reduces EMT, indicating that DSCR8/miR-98-5p modulates EMT and affects OC progression.

The JAK/STAT signaling pathway is one of the earliest discovered signaling pathways that regulate tumor progression. STAT3 activation is closely correlated with the tumorigenesis and chemotherapy and radiation tolerance of tumors, including OC (29, 30). Particularly, STAT3 activation promotes EMT of cancer cells (31). Moreover, the role of HIF-1α is also extensive in tumors. For example, HIF-1α is upregulated in HCC and strengthens its metastasis and vasculogenic mimicry (VM) formation by upregulating LOXL2 (32). Moreover, HIF-1α enhances the invasion and migration of OC cells induced by hypoxia (33). Interestingly, STAT3 promotes the expression of HIF-1α as its upstream molecule (17, 34). Here, we found that overexpressing DSCR8 greatly promoted the activation of STAT3/HIF-1α, while overexpressing miR-98-5p reduced STAT3 expression and activation, indicating that DSCR8/miR-98-5p regulates OC development by regulating STAT3/HIF-1α pathway.

On the other hand, as important transcription factors in cells, STAT3 and HIF-1α regulate the expressions of non-coding RNAs including lncRNAs and miRNAs. For example, the STAT3 pathway affects the spheroid formation and deterioration of OC by regulating miR-92a/DKK1-regulatory pathways (35). In addition, HIF-1α and lncRNA CASC9 form a positive feedback regulatory loop to regulate cell proliferation and metastasis in non-small-cell lung cancer (NSCLC) (36). We have affirmed the role of DSCR8/miR-98-5p/STAT3/HIF-1α axis in OC. Further, we used drugs to interfere with STAT3 and HIF-1α activation. Interestingly, we discovered that both HIF-1α and DSCR8 were markedly downregulated after inhibiting STAT3. In contrast, only DSCR8 was downregulated, while STAT3 activation was not distinctly affected after HIF-1α intervention. The results showed that there is a positive feedback loop, namely, DSCR8/miR-98-5p/STAT3/HIF-1α in OC progression.

## Conclusion

This study combined clinical and basic researches and confirmed that DSCR8 functions as an oncogene in OC and regulates the proliferation, metastasis, and apoptosis of OC through a positive feedback loop of DSCR8/miR-98-5p/STAT3/HIF-1α. In conclusion, this study provides new insights into the molecular mechanism of OC progression and is expected to bring more references for targeted treatment of OC.

## Data Availability Statement

The raw data supporting the conclusions of this article will be made available by the authors, without undue reservation.

## Ethics Statement

This study was approved by the Medical Ethics Committee of The First Affiliated Hospital of Harbin Medical University, and written informed consent was obtained from all patients involved in the study. The animal study was approved by the Ethics Review Board of The First Affiliated Hospital of Harbin Medical University.

## Author Contributions

DZ conceived and designed the experiments. LD and XC performed the experiments. YL and GZ performed statistical analysis. LD wrote the manuscript. All authors contributed to the article and approved the submitted version.

## Conflict of Interest

The authors declare that the research was conducted in the absence of any commercial or financial relationships that could be construed as a potential conflict of interest.

## Footnotes

1 http://kmplot.com/analysis/index.php?p=service

2 http://carolina.imis.athena-innovation.gr/diana_tools/web/index.php?r=lncbasev2%2Findex
